# Media coverage of COVID-19 vaccination-associated cerebral venous sinus thrombosis was followed by a surge in emergency presentations due to headache – observations from a university hospital in Germany

**DOI:** 10.3389/fpsyt.2024.1378472

**Published:** 2024-05-23

**Authors:** Livia Asan, Julian Kleine-Borgmann, Bessime Bozkurt, Benedikt Frank, Martin Köhrmann, Christoph Kleinschnitz, Ulrike Bingel

**Affiliations:** Department of Neurology and Center for Translational Neuro- and Behavioral Sciences (C-TNBS), University Hospital Essen, Essen, Germany

**Keywords:** headache, media, emergency care, side effects, COVID-19, vaccination, expectation, nocebo

## Abstract

Nocebo effects describe all negative outcomes for well-being brought about by negative health-related expectations. Media coverage of drug side effects can fuel nocebo effects and lead to increased symptom reports. This retrospective observational analysis of emergency reports at the neurological emergency room at University Hospital Essen, Germany, examines whether media communication about a cumulation of very rare cases of cerebral venous sinus thrombosis (CVST) after COVID-19 vaccination with the AstraZeneca compound (ChAdOx-1 nCoV-19) was followed by an increase in weekly presentation rates of patients with the main complaint of headache, a symptom commonly occurring as a vaccination reaction but also communicated as a warning symptom for CVST. The rate of headache presentations increased by 171.7% during the five weeks after the first announcement of CVSTs in Germany on 11 March 2021, compared to the five weeks immediately prior. Furthermore, more young women sought consultation for headache, reflecting the communicated at-risk profile for CVST. The increased rate of headache presenters contributed to a 32.1% rise in total neurological emergency cases, causing an increased strain on the emergency facility after the side effect risk was publicized. We discuss a causal role of negative side effect expectations after vaccination with AstraZeneca as a driver for this increase. While transparent communication about benefits and potential side effects is crucial for vaccination acceptance, increased vigilance toward nocebo effects in health-related media communication is needed due to its potential harm to the individual and society, especially when emergency medical resources are stretched thin.

## Introduction

Expectations can impact the perception of symptoms and substantially modulate treatment outcome. While the effects of positive expectation, also known as placebo effects, are desirable to optimize health outcomes as a component of an active treatment, their negative counterpart, i.e. nocebo effects, can elicit or worsen symptoms commonly resembling known side effects. Although the neural foundation of nocebo effects is less well understood than that of placebo effects, brain imaging studies have revealed brain activity changes in networks linking attention, anxiety and sensory processing, as well as changes in neurotransmitter systems (1). Mediators of negative expectations include verbal suggestions delivered by health care professionals or through information leaflets, social observation, previous treatment experience, beliefs about one’s own vulnerability, and the public perception of health risks (1). For instance, during a health scare now known as the Flint water crisis, a broad media proclamation of hazardous lead contamination in drinking water is now thought to have caused substantial suffering and societal burden via nocebo effects, which by far exceeded the extent of what could reasonably be explained by the measured lead levels alone (2).

Experimental studies have shown that headaches can be subject to strong nocebo effects induced merely by informing about the risk of headache occurring upon a certain exposure (3, 4). Alerting the public about headache as a side effect of a treatment leads to an increase in reports of headache, even when an inert placebo is applied (5). The relevance of nocebo effects in preventive treatments is apparent in the case of vaccinations. An analysis of large randomized controlled trials testing COVID-19 vaccines demonstrated that headache was reported by 19% of placebo recipients (6). Importantly, nocebo effects after vaccination can be induced through media coverage of side effects. For instance, in a study investigating adverse event reports after vaccination with Gardasil against the human papilloma virus, the number of news reports on Gardasil side effects significantly predicted the number of adverse event reports in the following month (7).

During the COVID-19 pandemic, media outlets received exceptionally high levels of attention due to the constant flux of information on the spread of the virus and restrictive containment measures affecting everyday life. When the first vaccine against COVID-19 from BioNtech/Pfizer (BNT162b2) was approved by the European Medicines Agency (EMA) in late December 2020^1^, stay-at-home restrictions and school and workplace closures were in place for all but key workers in Germany, and the number of confirmed deaths due to COVID-19 in Europe had reached a new peak^2^. EMA-approval of the COVID-19 vaccines by Moderna (mRNA-1273, 6 January 2021) and AstraZeneca (ChAdOx1nCoV-19, 29 January 2021) followed in close succession. Although the prospect of effective vaccines raised hopes for containing the virus and preventing severe diseases and deaths, there were reservations among the population regarding the safety of the vaccines due to their accelerated development and approval^3^. The launch of an extensive public vaccination campaign nevertheless resulted in 10.4 administered vaccine doses per 100 persons by 10 March 2021, in Germany, and vaccination rates further increased steadily in Germany over the following weeks^4^.

On 11 March 2021, first articles and news reports appeared in the German media informing about a temporary halt to vaccinations with the AstraZeneca compound AZD1222 (referred to as AstraZeneca vaccine in the following) in Denmark^5^. This was a safety precaution and a response to new information on seven cases of cerebral venous sinus thrombosis (CVST) that could potentially be linked to the vaccine. CVST is a cerebrovascular disease caused by a blood clot obstructing the venous blood drainage in the brain, which, in severe cases, can lead to cerebral edema and bleeding. Quickly picking up on this concern, headlines of influential German newspapers and radio outlets proclaimed “Thrombosis cases under investigation - doubts about the AstraZeneca vaccine”^6^, “Brain thromboses after AstraZeneca vaccination”^7^, and “Special thrombosis after vaccination primarily affects women”^8^. Television news reports featured experts informing about the symptoms and consequences of CVST, mentioning persistent headache as a symptom^9^. In the weeks following these first media announcements, worried patients reportedly “flooded” the neurological emergency rooms in Germany and elsewhere^10^ ^,^ ^11^, complaining of headache after COVID-19 vaccination and alarmed that their headache might be an indicator of the reported potentially life-threatening vaccine side effect. However, systematic studies analyzing emergency presentation rates due to headache in relation to this side effect information are lacking. Moreover, it remains unclear whether the awareness of the side effects also led, through an increased number of presentations to the emergency room due to headaches after vaccination, to the actual identification of CVST. Particularly in view of the challenges during the pandemic, when medical staff and resources were limited and focused on the care of critical patients, factors that potentially exert an unnecessary strain on individuals and the healthcare system need to be critically investigated. In this context, the potential role of the media as a driver of nocebo effects should be explored in order to optimize future risk communication and mitigate negative impact on individual health and society.

In the search for evidence to address these questions, we quantified the presentation rate of patients with the main complaint of headache in our neurological emergency room at the University Hospital in the city of Essen, Germany, before and after the CVST health risk was announced in the media. In alignment with previous studies investigating the role of media coverage on side effect reporting in a real-world setting (8, 9), we conducted a retrospective pre-post observational analysis, comparing weekly presentation rates of patients complaining of headache in our neurological emergency room during five weeks before and five weeks after the potential health risk was made public in Germany on 11 March 2021^12^ ^,^ ^13^. To confirm the population’s attention to and engagement with this topic in the five weeks after the media reports, we determined the number of Google searches matching the prompt “AstraZeneca cerebral vein thrombosis” in the region.

We hypothesized that weekly rates of patients presenting with headache to our neurological ER increased after the population was exposed to this media announcement. Using public data from the Robert Koch Institute (RKI)^14^, the German institution for epidemiology and nationwide health monitoring, we related the ER data to local COVID-19 vaccination rates in order to account for confounds by the increased number of vaccinations over time. Furthermore, we assessed whether the increase in weekly headache presentations resulted in higher total rates of neurological ER presentations, thereby estimating the impact on the overall utilization of our neurological emergency care.

In this way, our retrospective study aimed to quantify presentation rates with the primary complaint of headache in our neurological emergency room in relation to the media information about the risk of CVST after COVID-19 vaccination and to evaluate the evidence regarding potential nocebo-effects brought about by the media.

## Methods

### Study design

**Figure 1:**
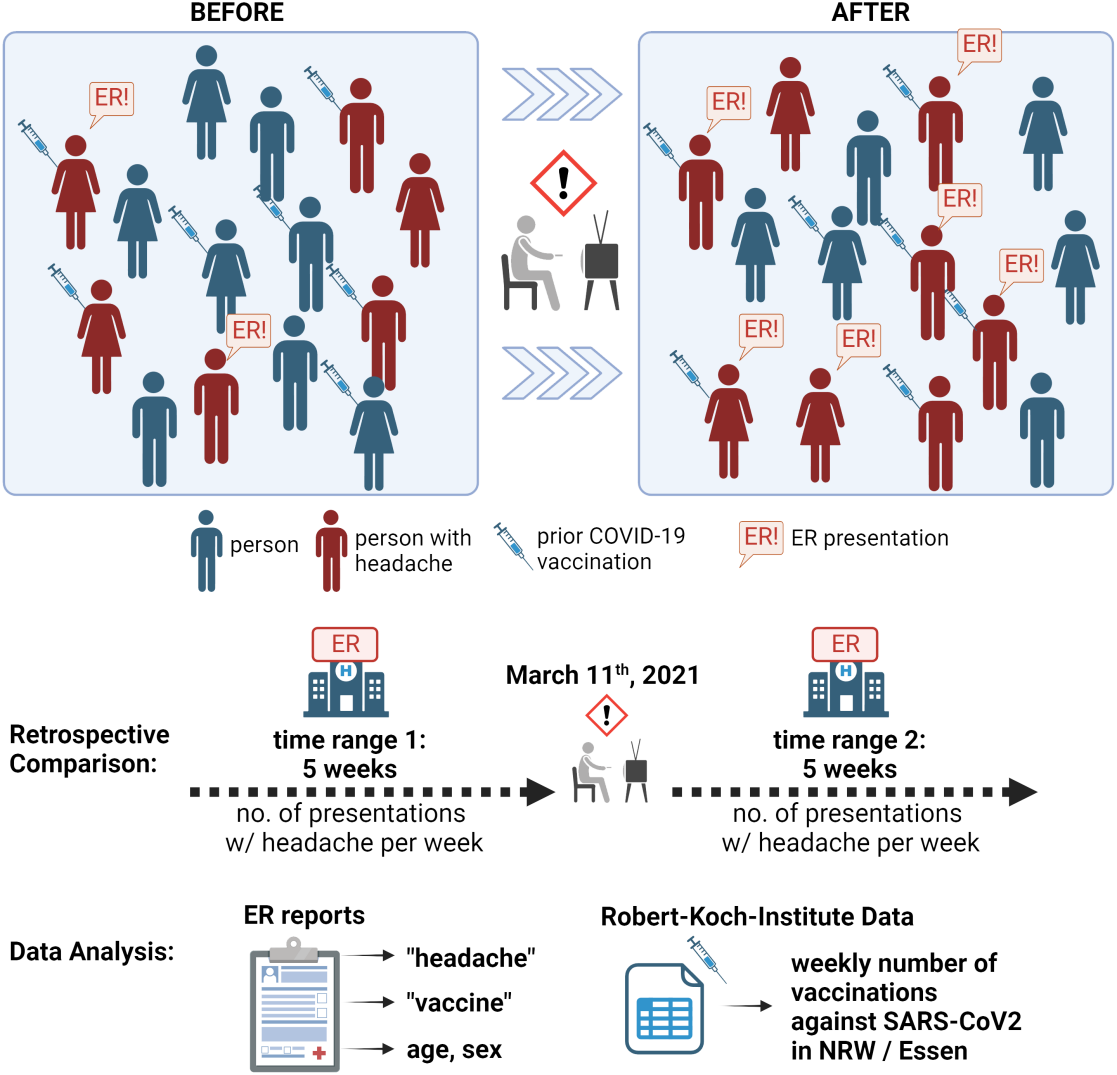
Schematic illustration of study data and extraction. Top section: The general population before and after media coverage of COVID-19-vaccination-related cerebral venous sinus thrombosis (television icon) are depicted schematically in the top section. Some people might experience headache (red-colored mannequins), some of them after prior vaccination (syringe icon). Importantly, the general incidence of headache (sum of red-colored mannequins) *cannot* be assessed with our data. However, a proportion of persons with headache present to the emergency room (ER, indicated with “ER!” speech bubble). In this study, we counted and analyzed all ER presentations due to headache that were registered in our department. Bottom section: ER reports during the five weeks before (time range 1, TR1) and after (time range 2, TR2) 11 March 2021, were analyzed in the present study. Search term screening was performed, and basic demographic characteristics (age, sex) were extracted. Information about vaccinations against COVID-19 in the area was obtained from a public repository provided by the Robert Koch Institute in Germany. NRW: Federal State of North Rhine-Westphalia. The illustration was created on BioRender.com.

This retrospective observational study analyzed data collected from all digital emergency reports recorded in the neurological emergency department at Essen University Hospital between 4 February and 14 April 2021. Reports were assigned to one of two time ranges depending on the time of presentation: Time range 1 (TR1) covered the five weeks before 11 March 2021, when news about the vaccine side effects was first broadcast in the German and international media (**Figure 1**). This first time range was chosen as the comparison time range, in which the population was not exposed to this specific media content. Time range 2 (TR2) covered the five weeks from 11 March 2021, which marks the time with increased exposure to warnings about the severe vaccination side effect of CVST. We chose to study five-week periods as clinical staff perceived the increase in headache presentations to be most pronounced in the first weeks following the news reports. We adhered to the STROBE guidelines for reporting observational studies (10).

### Data collection and processing

The data source is the hospital’s fully digitalized emergency documentation system ‘ERPath’ (epias GmbH, Team ERPath, Berlin, Germany). In this system, all neurological emergency presentations, i.e., all individual ER consultations, are routinely registered and documented in single emergency reports, irrespective of the route of presentation (by foot, by ambulance, by transfer from another hospital etc.). These reports include an individual presentation ID, patient ID, date of birth, sex, date and time of presentation, as well as free text fields for notes by the physician attending to the case, which routinely include diagnoses, patient history, examination results, assessment and treatment plan. Note that some patients presented to the ER several times, leading to several registered and counted presentations. The data for this study were extracted from ERpath on 31 January 2022, and consisted of all presentation reports of the neurological emergency room at Essen University Hospital between 4 February and 14 April 2021. From these, as the primary outcome, we counted the number of presentations per week in which headache was the main complaint (“headache presentations”), which was compared across TR1 and TR2. We chose to focus our analyses on weekly presentation rates since this is an easily intelligible number that lends itself to potential comparison at other sites. As a secondary outcome of interest, we also quantified the number of presentations which concluded with the diagnosis of CVST. For further exploration, we checked headache presentation reports for mentions of vaccinations and vaccine compounds used prior to presentation. We explored differences between the two time ranges in terms of age and sex of patients with headache presentations.

Data processing was tailored to identify headache presentation, diagnoses of CVST, and mentioned prior vaccinations among all neurological presentations. For this purpose, all text fields from the digital reports were automatically screened using predefined search terms in R (R version 4.0.4; RStudio 2023.09.1 Build 494). A presentation was classified as a positive hit whenever one or more search terms were present in the text. Asterisks indicate wildcards and search strings were case insensitive. The search strings loosely translate to the following English terms: “headache” OR “head pressure” OR “vacc” OR “Astrazeneca” OR “astra” OR “Zeneca” OR “Biontech” OR “Pfizer” OR “Moderna” OR “sinus*thrombosis” OR “cerebral*thrombosis” (original German strings: “Kopfschmerz” OR “Kopfdruck” OR “impf” OR “Astrazeneca” OR “Astra” OR “Zeneca” OR “Biontech” OR “Pfizer” OR “Moderna” OR “Sinus*thrombose” OR “Hirn*thrombose”). All reports of positive screening hits were then read thoroughly by trained medical personnel, who were blinded concerning the time point of presentation. The final inclusion criterion for a “headache presentation” was confirmation of headache as the main complaint leading to presentation. Exclusion criteria were i) headache reportedly associated with prior head trauma; ii) report of heavily impaired cognition or altered consciousness that precluded direct history taking from the patient. A “CVST presentation” was defined by the diagnosis of CVST as concluded in the ER report. All headache and diagnosed CVST presentations were further assessed for age, sex, any reported history of prior vaccination, and the vaccine used.

To account for a potential confound of increased headache presentations by higher vaccination rates against COVID-19, weekly vaccination rates as monitored by the RKI were obtained from Github^14^ for the city of Essen and the federal state of North Rhine-Westphalia (NRW), where Essen is located. We also screened data of ER reports during the time of “booster” vaccinations (= third vaccination against COVID-19), which was approximately 9 months after our main time range of interest. The ten weeks around 21 November 2021, are suitable for comparison since they also cover a time when many people got vaccinated against COVID-19 within a short period of time (see **Supplementary Figure 1**). We applied ANOVA to compare the number of hits in our screening for headache cases of all four of the five-week-time ranges (time range 1: 4 February - 10 March 2021; time range 2: 11 March – 14 April 2021; time range 3: 17 October - 20 November 2021, time range 4: 21 November – 27 December 2021). Additionally, we compared the variances of weekly screening hits with Levene’s test to check for stability of variances across all screened time ranges. Search volume data on Google were downloaded from Google Trends on 18 December 2023. Weekly search rates for “AstraZeneca cerebral vein thrombosis” (German: “AstraZeneca Hirnvenenthrombose”) in NRW were calculated and used as a proxy for the population’s reaction to media reports and attention to the topic.

### Statistical analysis

Data were tested for normal distribution using the Shapiro-Wilk-Test, and parametric or non-parametric statistical tests were chosen accordingly. Differences in weekly presentation rates and patients’ age between TR1 and TR2 were assessed using t-test or Wilcoxon test, respectively, and patients’ sex was compared using Chi Square test. To explore whether weekly headache presentation rates could explain the number of all weekly neurological presentations and whether this association was modulated by the time range, we calculated an ANOVA to explain the variance in weekly total neurological presentations by the factors of TR and weekly headache presentations as well as their interaction (ANOVA: weekly total neurological presentations ~ time range * weekly headache presentations).

To account for a potential confound on weekly headache presentations, we analyzed the variance of weekly headache presentations explained by the factor of time range and the weekly vaccination rates in Essen with i) any of the COVID-19 vaccines or ii) only the AstraZeneca vaccine as covariate (ANOVA: weekly headache presentations ~ time range * weekly vaccinations).

R version 4.0.4 was used to compute statistics and to create data plots (R version 4.0.4; RStudio 2023.09.1 Build 494).

## Results

**Figure 2:**
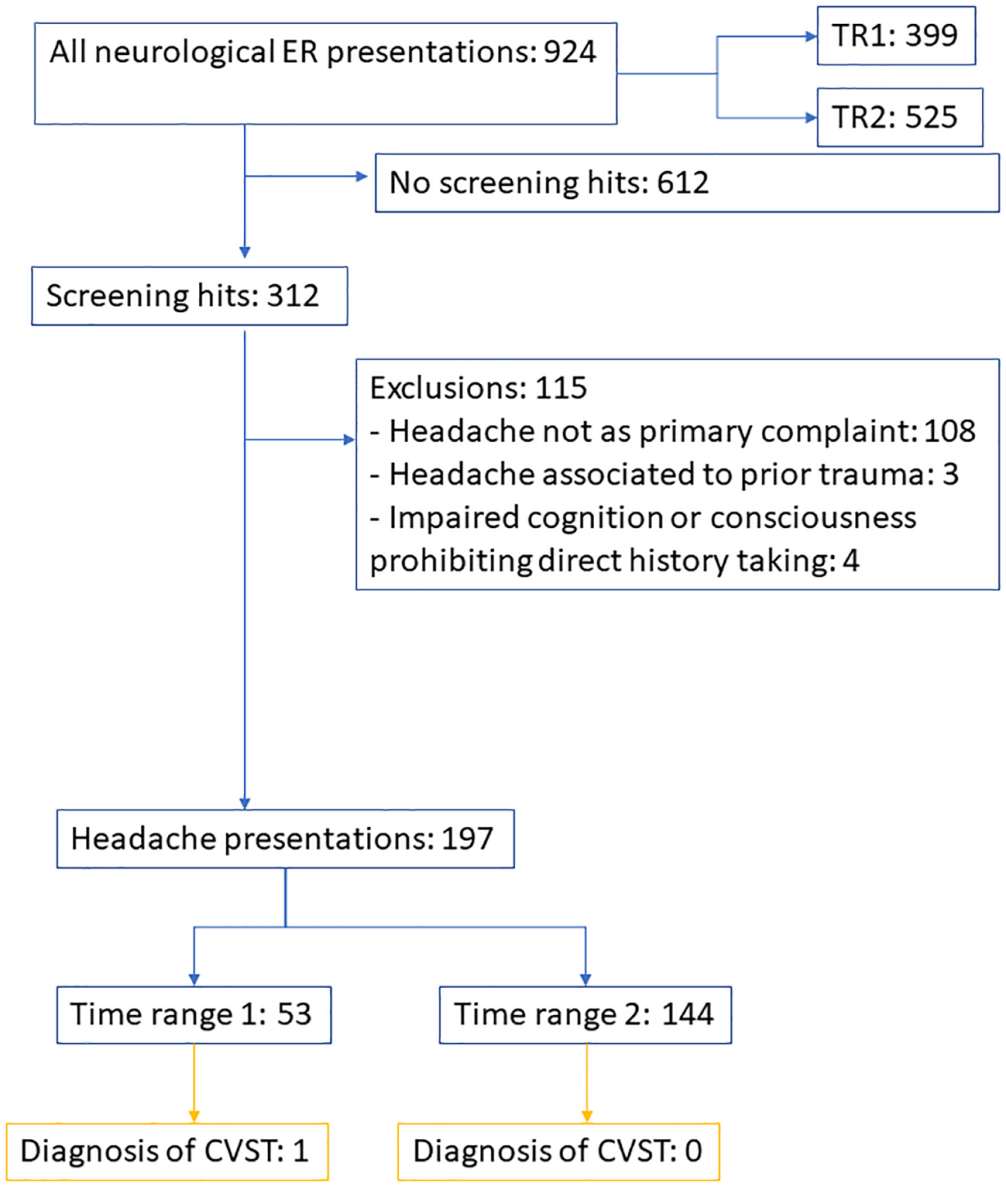
Flow chart displaying screening and inclusion for the number of headache presentations (primary outcome) and presentations with diagnosis of CVST (secondary outcome). TR, time range. CVST, cerebral venous sinus thrombosis.

**Figure 2** displays the number of neurological ER reports which entered screening and were checked for inclusion as headache presentations as the primary outcome, as well as for the diagnosis of CVST as the secondary outcome.

A total of 924 presentations were registered in the neurological ER in the ten weeks examined, of which 399 occurred during the first time range (TR1, spanning the five weeks before health risk media coverage), and 525 during the second time range (TR2, spanning the five weeks after health risk media coverage). Of the 924 presentations, 197 presentations were for patients reporting headache as the primary complaint and cause of consultation, of which 53 occurred in TR1 (constituting 13.3% of all neurological presentations during TR1) and 144 occurred in TR2 (constituting 27.4% of all neurological presentations during TR2). One of the headache presentations in TR1 was concluded with the diagnosis of cerebral sinus venous thrombosis, with no prior vaccination documented. No included case in TR2 was diagnosed with CVST. **Table 1** summarizes the demographic characteristics of presenters separately by time range.

**Table 1:** Data of patients and presentations during the studied time ranges.

	TR1 (weeks 1-5)	TR2 (weeks 6-10)	total 10 weeks
**All neurologic ER patients**	386	490	867*
Age (in years, ± IQR)	58.1 ± 35.0	53.5 ± 35.5	55.5 ± 36.0
Sex (female/male)	209/ 177	286/204	489/378
Presenting once	373	457	814
Presenting twice	13	31	49
Presenting three times	0	2	4
Number of presentations	399	525	924
**Headache ER patients**	50	133	183
Age (in years, ± IQR)	46.0 ± 27.2	37.6 ± 19.0	39.9 ± 22.0
Sex (female/male)	33/17	99/34	132/51
Presenting once	47	122	169
Presenting twice	3	11	14
Presenting three times	0	0	0
Number of presentations	53	144	197

Numbers of patients constituted the sample size for the comparison of presenters’ age and sex, while the number of presentations from these patients constituted the main outcome for this study to adequately assess utilization of ER resources. TR, time range. IQR, Interquartile Range.

*10 patients presented during TR1 and TR2, therefore, the sum of TR1 and TR2 exceeds the number of patients within the total 10-week period.

**Figure 3:**
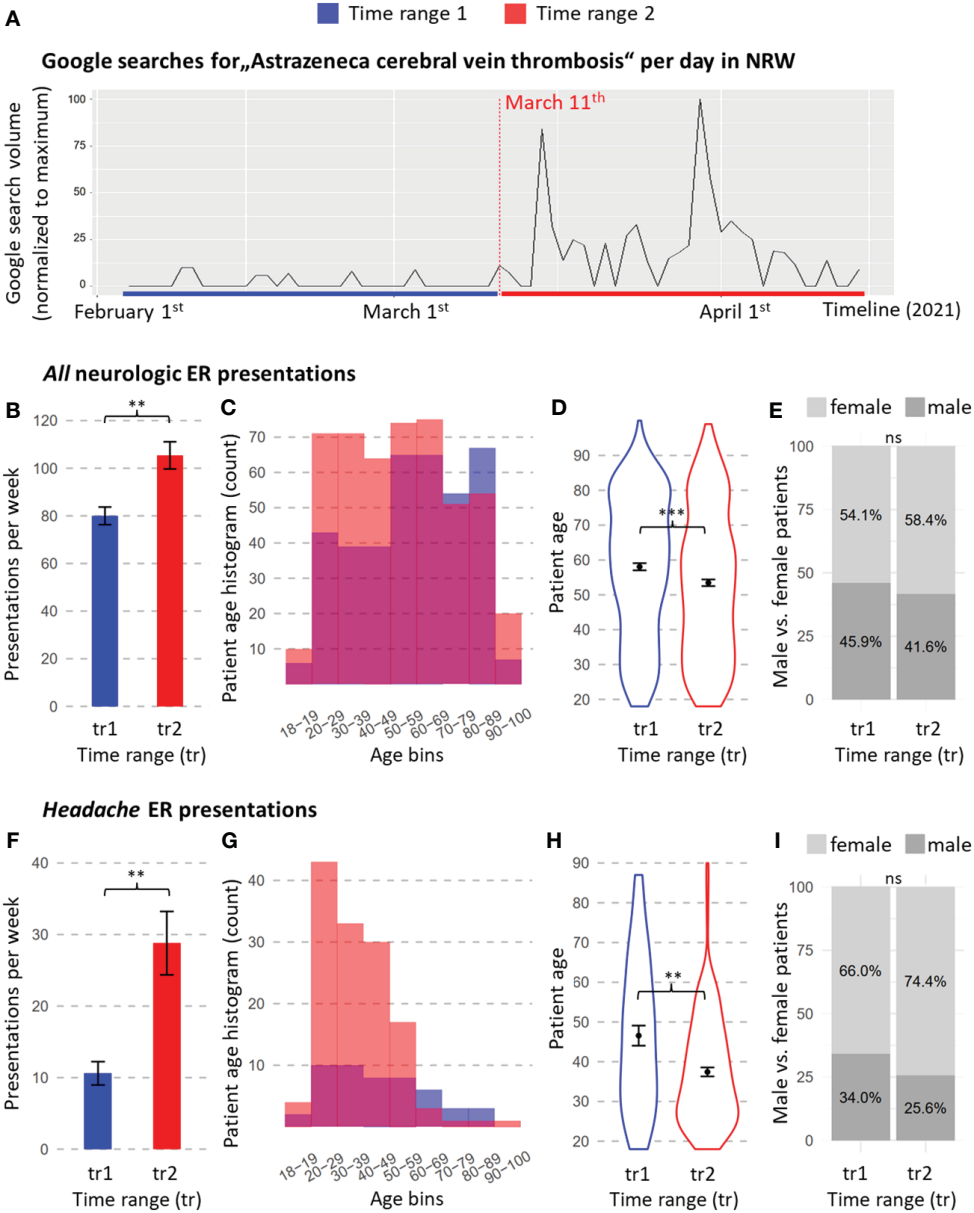
Internet searches in North Rhine-Westphalia (NRW) and data from digital emergency reports from the Neurology Department at University Medicine Essen. **(A)** Daily Google search volumes matching the search prompt “AstraZeneca cerebral vein thrombosis” (German: “AstraZeneca Hirnvenenthrombose”) as given by Google trends throughout both time ranges. Search counts are normalized to the maximum per day, representing a value of 100. Data for all neurological emergency reports: **(B–E)** data for headache presentations only: **(F–I)**. **(B, F)** Weekly presentation rates. **(C, G)** Histogram of patient age at the time of presentation to the ER. **(D, H)** Age distribution of presenting patients. **(E, I)** Percentages of female and male presenters. Significance codes: ‘***’ = p-value < 0.001; ‘**’ = p-value < 0.01; ‘*’ = p-value < 0.05. ns, non- significant. Means and standard errors of the means are shown with error bars. TR, time range. TR1 = 5 weeks before CVST news reports (4 February 2021 – 10 March 2021); TR2 = 5 weeks after CVST news reports (11 March 2021 – 14 April 2021).

The analysis of data on Google search volumes revealed a noticeable increase in Google searches with the terms “AstraZeneca” and “CVST” in NRW during TR2. Weekly search volumes were significantly higher in TR2 compared to TR1 (t-test, p= 0.003, **Figure 3A**).

The investigation of weekly presentation rates of all neurological ER presentations revealed 80.0 ± 3.7 presentations per week during TR1 and 105.4 ± 5.7 presentations per week during TR2. This increase of 32.1% was significant (two-sided t-test: p = 0.003; **Figure 3B**). When focusing on presentations with the leading symptom headache, the presentation rates rose from 10.6 ± 1.6 per week during TR1 to 28.8 ± 4.4 per week during TR2; this difference was statistically significant (two-sided t-test: p=0.002, **Figure 3F**), and represents a 171.7% rise in headache presentations from TR1 to TR2. The number of headache presentations per week explained the number of total neurological presentations per week across both time ranges, with no main or modulatory effect of time range (factor of weekly headache presentations: F = 11.886, p = 0.014, eta squared = 0.054, partial eta squared = 0.148; factor of TR: F = 1.431, p = 0.277; interaction of TR and weekly headache presentations: F = 0.013, p = 0.914), suggesting that the rise in weekly headache explained the increase in weekly total neurological presentations.

The age distribution of patients presenting to the neurological ER shifted toward younger patients, from 57.9 ± 1.0 years in TR1 to 53.4 ± 0.9 years in TR2 (two-sided t-test: p=0.0008, **Figures 3C, D**). When limiting the analyses to headache presentations, a more pronounced drop in age emerged, from a mean of 46.6 ± 2.5 years in TR1 to a mean of 37.6 ± 1.1 years at TR2 (Wilcoxon test: p= 0.002, **Figures 3G, H**).

Slightly more females presented to the neurological ER in both time ranges (54.1% in TR1, 58.4% in TR2), with no statistically significant difference between the time ranges (p = 0. 237, **Figure 3E**). Headache presenters were more often female (66.0%) than male (**Figure 3I**) in TR1, and the proportion of women increased further in TR2 (74.4%). A Chi-Square test showed that this difference in headache presenters’ sex was not statistically significant (p=0. 343, **Figure 3H**).

All headache presentations were checked for a documented history of vaccination. During TR1, a total of two prior vaccinations were reported: one against measles and one against COVID-19 with the BioNTech vaccine. The remaining 51 charts did not mention vaccinations or denied prior immunization. In TR2, 80 presentations documented prior vaccination against COVID-19 with AstraZeneca; three with the BioNTech vaccine, three with the Moderna vaccine, and 58 did not mention or deny previous vaccination.

**Figure 4:**
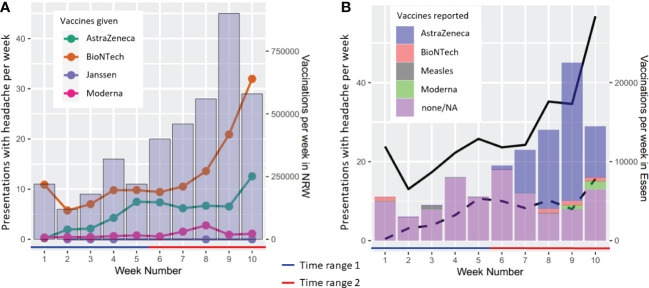
Weekly presentation rates due to headache in relation to vaccination rates in the German state of North Rhine-Westphalia (NRW) and the city of Essen. **(A)** Headache presentation rates and vaccinations per week registered in the state of NRW, separated by the manufacturer (color-coded). **(B)** Headache presentation rates and vaccinations per week in Essen. Solid line: Total COVID-19 vaccinations registered in the district of Essen. Dashed line: approximated AstraZeneca vaccinations in Essen. Calculation based on the weekly proportion of AstraZeneca vaccines used in NRW, which were then related to the sum of vaccinations per week in Essen.

Over the analyzed period, the rates of vaccinations against COVID-19, including immunizations with the compound by AstraZeneca, increased over time in Essen and NRW (**Figure 4A**). Therefore, a rise in headache presentations reaching our ER may potentially be entirely explained by a proportional increase in patients presenting with headache as a common vaccination reaction. We aimed to account for this potential confound by analyzing the variance of weekly headache presentations explained by the TR and by including information on weekly vaccination rates in Essen with any of the COVID-19 vaccines or only the AstraZeneca vaccine (**Figure 4B**) as a covariate. The results revealed no significant main effect of vaccination (all vaccines: F = 0.893, p = 0.381; AstraZeneca only: F = 0.016, p = 0.904) and no significant interaction between TR and vaccination (all vaccines: F = 0.106, p = 0.756, AstraZeneca only: F = 0.086, p = 0.779), whereas TR significantly explained weekly headache presentations (all vaccines: F = 13.868, p = 0.010, eta squared =0.260, partial eta squared = 0.475; AstraZeneca only: F = 12.090, p = 0.0132, eta squared = 0.323, partial eta squared = 0.495).

In order to provide evidence that the rise in weekly headache presentations observed after the media coverage is unlikely to be attributed to natural fluctuations of this outcome measure over time, we examined the number of screening hits per week also within two time ranges during the booster vaccination period, with a similar count of vaccinations against COVID-19, but no recent media warning about side effects (TR3: five weeks between 17 October - 20 November 2021, and TR4: five weeks between 21 November – 27 December ). Indeed, the number of screening hits per week during TR3 and TR4 did not differ from TR1, whereas TR2 differed from all other time ranges, and the variance of weekly screening hits was stable across all time ranges (see **Supplementary Figure 2**).

## Discussion

In summary, our data showed a 172% increase in weekly headache presentation rates in the five weeks following news coverage of the risk of CVST after immunization with AstraZeneca. Several of our findings hint at a substantial role of media-induced nocebo effects in this drastic increase in headache presentation rates.

First and foremost, the increase was not explained by the rising numbers of vaccinations in Essen or NRW during that time. Furthermore, comparing the numbers of screening hits with those during the period of booster vaccinations showed that only the time range after the media coverage was accompanied by a rise in screening hits. Hence, the increased presentations to our ER were largely independent of the mere increase in exposure to the drug but were significantly higher after the media coverage. Querying data from Google, we confirmed that Google searches matching the terms “AstraZeneca cerebral vein thrombosis” markedly increased after the first reports of an association on 11 March 2021, confirming swift surge of interest in and heightened attention to this health risk among the population. Furthermore, patients presenting to the ER with headache were significantly younger following the media coverage than in the time range immediately prior to it, and we observed a trend that more women in particular sought consultation. These findings correspond to the media coverage of risk profiles, which attributed the highest risk of CVST after vaccination to young females^15^, further strengthening the notion that individual expectations of vulnerability to side effects played a role. At this point, it is important to note that only one of all included headache presentations was concluded with the diagnosis of CVST, and this occurred during TR1, with no information about prior vaccination stated in the chart. Accordingly, presenting to the ER with headache as the primary complaint was *not* associated with an increased detection rate of CVST in TR2, even if prior vaccination was reported.

### Limitations of the study

Notably, some methodological considerations limit the interpretation of causality between media communication of the risk and the experience of headache after vaccination. Our analysis relied entirely on ER reports from patients who sought emergency consultation in our department, and a substantial reporting bias underlying our data must be suspected. We cannot make any direct inferences about a rise in the occurrence of headache after vaccination in the general population due to media-induced nocebo effects since we did not sample from the general population. Theoretically, it is possible that the proportion of vaccine recipients experiencing headache actually remained the same before and after the media announcement, and that merely the fraction of people who decided to present to our ER with these symptoms increased. However, if this was the case, it would still indicate that a frequent and primarily harmless symptom such as headache as a vaccination reaction was suddenly perceived as much more threatening than before, prompting people to seek emergency consultation. Anxiety and fear of side effects are known psychological factors associated with the generation and strength of nocebo effects (11, 12), which is why it is likely that at least in some cases, the symptom of headache was induced or aggravated by nocebo mechanisms.

### Comparison with the literature

Evidence from previous work further corroborates a key role of nocebo mechanisms in the present findings. First, we know from the large clinical trials on the efficacy and safety of vaccines against COVID-19 that side effects such as headache appeared frequently in the placebo-treated cohort, representing large nocebo responses (6). Second, experimental (13) and real-world evidence (7, 8) strongly suggests a crucial role of news coverage in symptom generation. In the communication of health risks, television coverage seems to be especially impactful in eliciting nocebo effects (14). Third, surveys investigating social media consumption showed that the number of posts seen about COVID-19 side effects, and the severity of symptoms conveyed in the posts, were predictive of the severity of side effects experienced after vaccination, indicating a “dose-dependent” effect of media exposure (15, 16).

## Conclusion

Our results highlight the effect of broad media risk communication on the utilization of emergency care facilities, with indications that a media-induced nocebo effect contributes to this phenomenon. Experts have put forward recommendations on how to mitigate nocebo effects in health risk communication (17), which should be considered as a basis for further scientific testing. In the case of vaccinations, these include a focus on the personal and societal benefits of a vaccination and a positive framing of mild side effects as a sign of a responsive immune system. Furthermore, misinformation should be addressed, and precautionary measures such as the temporary suspension of a vaccine should be emphasized as a clear indicator that functioning control mechanisms are in place, securing ongoing and rigorous safety assessments as cornerstones of high-quality modern, evidence-based medicine. Politicians, health institutions, and the media are responsible for acknowledging the medical and societal consequences of their communication and should endorse scientific efforts to find evidence-based strategies to reduce nocebo effects while fulfilling their duty to transparently inform the public.

## Data availability statement

The raw data supporting the conclusions of this article will be made available by the authors, without undue reservation.

## Ethics statement

The studies involving humans were approved by Ethics Committee of the Medical Faculty of the University of Duisburg-Essen (21-10365-BO). The studies were conducted in accordance with the local legislation and institutional requirements. Written informed consent for participation was not required from the participants or the participants’ legal guardians/next of kin because anonymized data from clinical routine were analyzed retrospectively.

## Author contributions

LA: Conceptualization, Data curation, Formal analysis, Investigation, Methodology, Visualization, Writing – original draft. JK: Conceptualization, Methodology, Writing – review & editing. BB: Data curation, Writing – review & editing, Investigation. BF: Writing – review & editing, Data curation, Methodology. MK: Resources, Writing – review & editing, Project administration. CK: Resources, Supervision, Writing – review & editing, Project administration. UB: Writing – review & editing, Conceptualization, Funding acquisition, Project administration, Resources, Supervision.

## Footnotes

1 European Medicines Agency. https://www.ema.europa.eu/en/human-regulatory-overview/public-health-threats/coronavirus-disease-covid-19/covid-19-medicines [Accessed December 17, 2023].

2
https://ourworldindata.org/coronavirus [Accessed December 17, 2023].

3 Imperial College London. https://www.imperial.ac.uk/media/imperial-college/institute-of-global-health-innovation/GlobalVaccineInsights_ICL-Covid-19-Behaviour-Tracker-EMBARGOED-00.01-04.02.2021.pdf [Accessed January 2, 2024].

4
https://ourworldindata.org/coronavirus/country/germany#how-many-covid-19-vaccine-doses-have-been-administered-in-total [Accessed December 17, 2023].

5 Spiegel. (March 11,2021). https://www.spiegel.de/wissenschaft/medizin/astrazeneca-daenemark-setzt-coronaimpfungen-vorsichtshalber-aus-a-b6d1e7c1-2164-4648-a2e7-7b0bfdc513a3 [Accessed November 5, 2023].

6 Frankfurter Allgemeine Zeitung. (March 15, 2021). https://www.faz.net/aktuell/politik/inland/zweifel-an-astrazeneca-impfstoff-blutgerinnsel-in-hirnvenen-17245969.html [Accessed December 17^th^, 2023].

7 SWR Wissen. (March 16, 2021). https://www.swr.de/wissen/corona-impfung-mit-astra-zeneca-in-deutschland-vorlaeufig-gestoppt-100.html [Accessed December 17, 2023].

8 Zeit online. (March 16, 2021). https://www.zeit.de/wissen/gesundheit/2021-03/astrazeneca-corona-impfstoff-thrombose-frauen-aussetzung [Accessed December 17, 2023].

9 Tagesschau. (March 16, 2021). https://www.tagesschau.de/inland/gesellschaft/astrazeneca-faq-101.html [Accessed December 17, 2023].

10 Westdeutsche Allgemeine Zeitung. (March 24, 2021). https://www.waz.de/staedte/essen/astrazeneca-anstieg-von-kopfschmerz-patienten-in-essen-id231880993.html [Accessed November 5, 2023].

11 The Guardian. (April 10, 2021). https://www.theguardian.com/society/2021/apr/09/ae-swamped-with-patients-seeking-help-for-mild-covid-jab-side-effects [Accessed November 5, 2023].

12 Zeit online. (March 11, 2021). https://www.zeit.de/wissen/2021-03/corona-impfstoff-astrazeneca-daenemark-impfungen-stopp [Accessed November 5, 2023].

13 Spiegel. (March 11, 2021). https://www.spiegel.de/wissenschaft/medizin/astrazeneca-daenemark-setzt-coronaimpfungen-vorsichtshalber-aus-a-b6d1e7c1-2164-4648-a2e7-7b0bfdc513a3 [Accessed November 5, 2023].

14 Robert Koch Institut. https://github.com/robert-koch-institut/COVID-19-Impfungen_in_Deutschland [Downloaded from GitHub January 10, 2022].

15 Paul-Ehrlich-Institut. (April 16, 2021). https://www.pei.de/SharedDocs/Downloads/DE/newsroom/meldungen/faq-temporaere-aussetzung-astrazeneca.pdf?:blob=publicationFile&v=2 [Accessed November 5, 2023].

## Supplementary material

Supplementary Figure 1 Display of the number of cumulative vaccinations against COVID-19 during the main time ranges of interest (around 11 March 2021) and during the period of first booster vaccinations (around 21 November 2021). A similar number of vaccinations have been recorded on 11 March 2021 (6.1 million first vaccinations) and on 21 November 2021 (6.4 million first booster vaccinations), according to official data from the Robert-Koch-Institute, displayed on the COVID-19 vaccination dashboard of the Federal Ministry of Health in Germany (https://impfdashboard.de/), accessed on 3 April 2024. A quick rise in vaccinations can be observed around 11 March as well as November 21^st^. Showing a similar increase in vaccination rates, both time periods can be compared to assess the stability in the number of screening hits for the number of ER presentations due to headache per week and thus to evaluate the specificity of the observed rise in headache presentations after the media coverage about CVST starting 11 March.

Supplementary Figure 2 Comparison of the screening hits per week for headache presentations during the main time range of interest and the booster vaccination period. Bar graph showing the mean and standard deviations for the number of screening hits per week during the “main” time ranges before (week numbers 1–5; time range 1) and after (week numbers 6–10; time range 2) 11 March 2021, and the “booster” time ranges before (week numbers 1–5; time range 3) and after (week numbers 6–10; time range 4) 21 November, 2021. ANOVA with *post-hoc* Tukey comparisons shows that weekly screening hits in in time range 2 differs from all other time ranges significantly. The other time ranges do not significantly differ from each other. Levene’s test revealed no difference in variances (p = 0.557) between all four time ranges. Significance codes: ‘***p-value < 0.001; ‘**p-value < 0.01; ‘*p-value < 0.05. ns = non- significant.
