# Supplementary figures and images for: Media coverage of COVID-19 vaccination-associated cerebral venous sinus thrombosis was followed by a surge in emergency presentations due to headache – observations from a university hospital in Germany
Source: Front Psychiatry. 2024 May 23;15:1378472. doi: 10.3389/fpsyt.2024.1378472 (PMC11153761; doi:10.3389/fpsyt.2024.1378472)

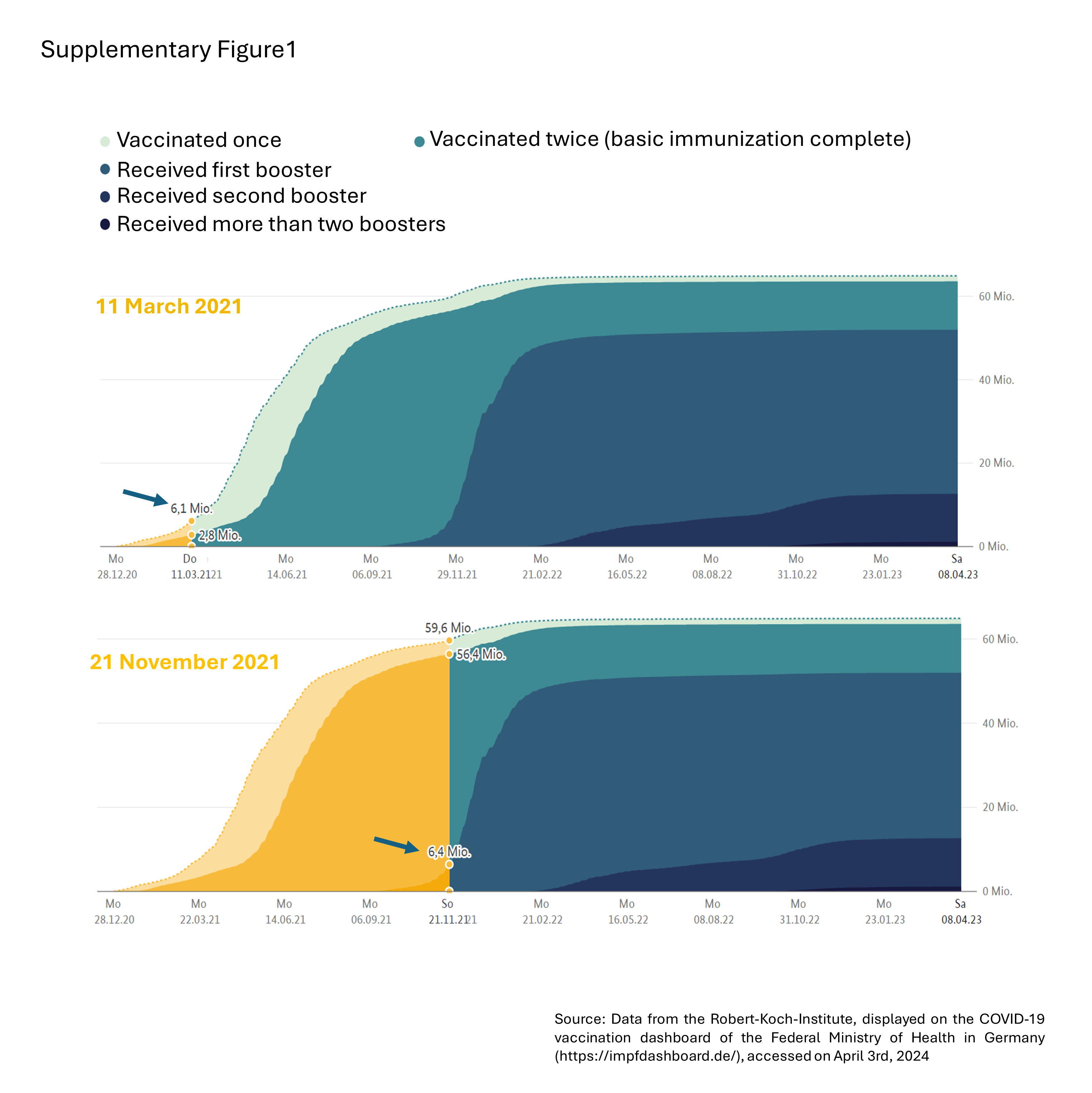

Supplement: Supplementary Figure 1 — Display of the number of cumulative vaccinations against COVID-19 during the main time ranges of interest (around 11 March 2021) and during the period of first booster vaccinations (around 21 November 2021). A similar number of vaccinations have been recorded on 11 March 2021 (6.1 million first vaccinations) and on 21 November 2021 (6.4 million first booster vaccinations), according to official data from the Robert-Koch-Institute, displayed on the COVID-19 vaccination dashboard of the Federal Ministry of Health in Germany (https://impfdashboard.de/), accessed on 3 April 2024. A quick rise in vaccinations can be observed around 11 March as well as November 21st. Showing a similar increase in vaccination rates, both time periods can be compared to assess the stability in the number of screening hits for the number of ER presentations due to headache per week and thus to evaluate the specificity of the observed rise in headache presentations after the media coverage about CVST starting 11 March. [file Image_1.tif]

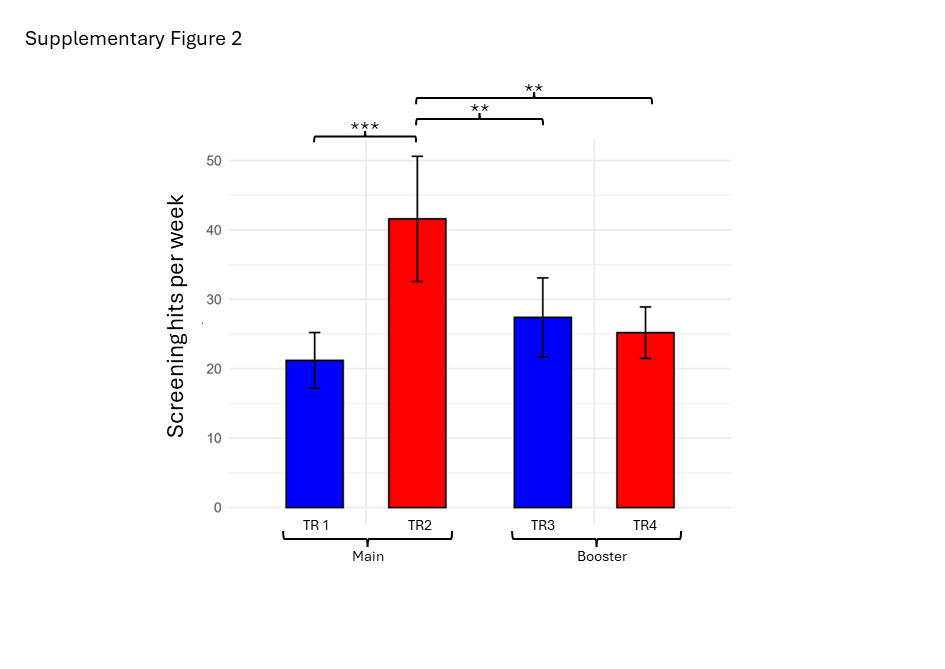

Supplement: Supplementary Figure 2 — Comparison of the screening hits per week for headache presentations during the main time range of interest and the booster vaccination period. Bar graph showing the mean and standard deviations for the number of screening hits per week during the “main” time ranges before (week numbers 1–5; time range 1) and after (week numbers 6–10; time range 2) 11 March 2021, and the “booster” time ranges before (week numbers 1–5; time range 3) and after (week numbers 6–10; time range 4) 21 November, 2021. ANOVA with post-hoc Tukey comparisons shows that weekly screening hits in in time range 2 differs from all other time ranges significantly. The other time ranges do not significantly differ from each other. Levene’s test revealed no difference in variances (p = 0.557) between all four time ranges. Significance codes: ‘***p-value < 0.001; ‘**p-value < 0.01; ‘*p-value < 0.05. ns = non- significant. [file Image_2.tif]
